# Psychoplastogens – a risk-benefit analysis and perspectives for future research

**DOI:** 10.1192/j.eurpsy.2025.2107

**Published:** 2025-08-26

**Authors:** V. A. Voicu, O. Vasiliu, A. Ciobanu

**Affiliations:** 1 Carol Davila University of Medicine and Pharmacy; 2 Romanian Academy of Medical Sciences; 3 Romanian Academy; 4 Psychiatry Dept., Dr. Carol Davila University Emergency Central Military Hospital; 5 “Prof. Al. Obregia” Psychiatry Clinical Hospital, Bucharest, Romania

## Abstract

**Introduction:**

The continuous exploration of new treatments in the field of psychopharmacology has brought a new light on psychedelics, raising the provocative question of how these drugs of abuse (DOA) may become useful in clinical practice. Psychedelics are included in the category of “psychoplastogens”, substances that are known for their effects of enhancing neuroplasticity in the nervous central system via the modulation of Brain-derived Neurotrophic Factor (BDNF) signaling. However, psychedelics were originally known as DOA; therefore, ascribing them to therapeutic use for patients with psychiatric disorders may seem largely counterintuitive.

**Objectives:**

To review the current data on the benefits and risks of psychoplastogens in patients with psychiatric disorders.

**Methods:**

A literature review was conducted in four electronic databases (PubMed, EMBASE, Cochrane, and Clarivate/Web of Science) and the US National Library of Medicine database for clinical trials (www.clinicaltrials.gov) to find clinical and preclinical sources published between January 2000 and September 2024. The keywords used were “psychoplastogens,” “neuroplastogens,” “neuroplasticity,” “psychoactive drugs,” “drugs in the pipeline,” and all the main psychiatric diagnosis categories. Both primary and secondary reports were allowed, but only those published in English were selected.

**Results:**

Ketamine and each of its stereoisomers, as well as psilocybin, are the most extensively explored drugs in this class, but also MDMA, DMT, psilocin (ELE-101), CYB003 (a psilocybin analog), and lisuride have received increased attention in the last decade. Such agents are investigated for indications such as treatment-resistant major depression, posttraumatic stress disorder, binge eating disorder, and substance use disorders. One important direction of research is the evaluation of psilocybin in patients with cancer-related depression and/or anxiety. Hallucinations and altered states of consciousness that may receive mystical interpretations are typical for high doses of psychedelics, raising questions about the use of these drugs in clinical populations with already severe mood, thought and perceptual disturbances. Safety and tolerability aspects are extremely important in deciding when, to whom, and how much psychoplastogens may be recommended for different psychiatric disorders. Creating psychoplastogens with less or no psychotomimetic activity is expected to increase the interest of clinicians in the use of such agents for patients with psychiatric disorders, especially in treatment-resistant cases.

**Conclusions:**

Although expected to be a paradigm-shifter in psychiatry, the exploration of psychoplastogens should consider not only the potential benefits, which require further and extensive studies, but also their adverse events. For this purpose, long-term studies are needed with both efficacy and tolerability outcomes carefully monitored.

**Disclosure of Interest:**

None Declared

